# Autoimmune Diseases in Patients with Cushing's Syndrome after Resolution of Hypercortisolism: Case Reports and Literature Review

**DOI:** 10.1155/2018/1464967

**Published:** 2018-12-18

**Authors:** Luigi Petramala, Federica Olmati, Maria Gabriella Conforti, Antonio Concistré, Valeria Bisogni, Nikita Alfieri, Gino Iannucci, Giorgio de Toma, Claudio Letizia

**Affiliations:** ^1^Secondary Hypertension Unit, Department of Transactional Medicine and Precision, University of Rome “La Sapienza”, Rome, Italy; ^2^Department of Surgery “P. Valdoni”, University of Rome “La Sapienza”, Rome, Italy

## Abstract

**Introduction:**

Cushing's syndrome (CS) is a clinical condition characterized by excessive cortisol production, associated with metabolic complications, such as diabetes mellitus, dyslipidemia, metabolic syndrome, hypertension, and cardiovascular diseases. Nowadays, the occurrence of autoimmune diseases in CS have not been completely evaluated in the previous studies.

**Objective:**

The aim of this study was to evaluate the occurrence of autoimmune diseases in CS patients after successfully treated.

**Materials and Methods:**

From January 2001 to December 2017, in our Secondary Hypertension Unit, we evaluated 147 CS patients (91 with ACTH-independent disease, 54 with ACTH-dependent disease, and 2 patients with ectopic ACTH production.

**Results:**

109 CS patients (74.1%) were surgically treated (67 ACTH-independent CS patients (61.5%) undergone adrenalectomy and 42 ACTH-dependent CS (38.5%) undergone transsphenoidal surgery) and evaluated after 6, 12, and 24 months after clinical and biochemical remission of disease. In 9 (8.3%) of overall treated CS patients (8.3%), during follow-up, we observed the onset of some manifestations of autoimmune diseases. In particular, one patient had a systemic lupus erythematosus, one patient had rheumatoid arthritis, 4 patients reported autoimmune thyroiditis (Basedow-Graves' disease and Hashimoto's thyroiditis), one patient had clinical features of psoriasis, one patient showed myasthenia gravis, and one patient had giant cell arteritis.

**Conclusions:**

Our results demonstrate that patients successfully treated for CS could develop autoimmune diseases. Therefore, after treatment, CS patients need to be strictly monitored in order to evaluate the possible onset of autoimmune diseases.

## 1. Introduction

Cushing's syndrome (CS) is a rare endocrine disorder characterized by prolonged, inappropriate excess of cortisol secondary to endogenous or exogenous causes [1]. Exogenous hypercortisolism is usually caused by prolonged administration of corticosteroids for chronic inflammatory diseases. On the other hand, endogenous hypercortisolism can be adrenocorticotropic (ACTH)-dependent or indipendent [2]. Overall, ACTH-dependent forms account for about 60–85% of cases, and of these, 80% are due to pituitary adenomas, whereas the remaining 20% are related to ectopic ACTH production. ACTH-independent CS is mostly due to adrenal tumors (adenoma in 60% and carcinoma in 40% of cases) [1]. The mainly described clinical features of CS are facial plethora, rounded face, decreased libido, thin skin, decrease linear growth in children, menstrual irregularity, glucose intolerance, hirsutism, easy bruising, osteoporosis, hypertension, and depression/emotional lability [3–5]. In addition, hypercortisolism is also characterized by its inhibitory function on immune system in humans [6]. In fact, several evidences shown that excess of cortisol production determines the involution of lymphoid tissue mass and lymphopenia with an increased susceptibility to infections [7]. The restoration of physiological production of cortisol can be associated to immune reaction, leading to the onset of autoimmune diseases [8]. In the literature, after hypercortisolism remission in CS patients, some case reports reported the onset of autoimmune diseases, such as Hashimoto autoimmune thyroiditis and Graves' disease [9]. In particular, the evidence on the development of autoimmune manifestations after surgical resolution of hypercortisolism is not well described and focused in clinical practice.

The aim of our study was to evaluate the occurrence of some newly secondary diagnosed autoimmune diseases in patients with CS after successful treatment.

## 2. Materials and Methods

From January 2001 to December 2017, we have retrospectively evaluated 147 CS patients (38 male (26%), 109 female (74%), mean age 52 years), referred to our Secondary Hypertension Unit, University of Rome “Sapienza,” Italy. Informed consent was obtained from all subjects. Of these 147 cases, 91 (61.9%) patients had an ACTH-independent CS, 54 (36.7%) patients were affected by an ACTH-dependent CS, and 2 (1.3%) patients with CS due to ectopic ACTH production.

In all groups, 66.6% CS patients underwent surgical treatment (67 (61.5%) patients with ACTH-independent syndrome underwent laparoscopic adrenalectomy and 42 (38.5%) patients with ACTH-dependent disease underwent transsphenoidal hypophyseal microneurosurgery), showing complete remission of cortisol excess based on the normalization of biochemical and clinical features during follow-up.

### 2.1. Cushing's Syndrome Diagnosis

The CS diagnosis was based on the clinical signs and symptoms (central redistribution of fat, hypertension, osteoporosis, and muscle weakness), hormonal data, and imaging tests (pituitary magnetic resonance imaging (MRI), adrenal computed tomography (CT), and adrenal scintiscan). Endocrine tests for CS showed a lack of diurnal rhythm of plasma cortisol (PC) levels (>138 nmol/L or >5 *μ*g/dL at midnight), no suppression of the PC levels (>50 nmol/L or >1.8 *μ*g/dL) after overnight 1 mg dexamethasone suppression test (DXM), and increased 24-hour urinary free cortisol (UFC) excretion (> 270 nmol/24 h or > 100 *μ*g/24 h). Measurement of plasma ACTH differentiated ACTH-dependent CS (ACTH ≥ 10 pg/mL) from ACTH-independent CS (< 10 pg/mL). ACTH was measured with the radioimmunoassay method (RIA), normal limit 10–90 pg/mL and inter/intra-assay CV 8.3/6.2, respectively; PC was measured with the radioimmunoassay method (RIA), normal limit 9.6–26 *μ*g/dL and inter/intra-assay CV 5.5/4.5, respectively; 24h urinary free cortisol was measured with the radioimmunoassay method (RIA), normal limit 1.37–7.53 *μ*g/24 h and inter/intra-assay CV 5.5/4.5, respectively. For ectopic ACTH-dependent differential diagnosis, patients underwent desmopressin test, corticotropin-releasing hormone (CRH) test, and inferior petrosal sinus sampling (IPSS) with desmopressin stimulus indicated when MRI was negative for a pituitary lesion; some patients with initial occult tumors underwent octreotide imaging (octreoscan) [10].

## 3. Results

After surgical removal of hypercortisolism, overall CS patients have been evaluated at 6, 12, and 24-month. During follow-up, nine out of them (8.25%) showed the onset of symptoms and signs of autoimmune diseases, previously not manifested (Table 1). Average time of clinical manifestation was 8.4 months, ranging from 4 up to 24 months. The ratio of female to male patients was 8 : 1. In particular, the autoimmune diseases were 1 patient with rheumatoid arthritis, 1 patient with systemic lupus erythematosus (SLE), 4 patients (3 women and 1 man) with autoimmune thyroid diseases, 1 patient with psoriasis, 1 patient with myasthenia gravis, and, finally, 1 patient with giant cell arteritis.

In Table 2, we reported the demographic, anthropometric, hemodynamic, biohumoral, and hormonal parameters of overall patients evaluated and the distinction of the two groups in relation to the development of autoimmune disease after surgical treatment (group A: patients without development of autoimmune disease; group B: patients with development of autoimmune disease).

Group A showed older age compared to group B (61.0 ± 11.7 yrs vs. 52.3 ± 12.7 yrs, *p* < 0.03).

Interestingly, at baseline, we found that group B showed significantly higher levels of PC and 24h-UFC compared to group A (818.1 ± 245.0 nmol/L vs. 459.5 ± 161.1 nmol/L, *p* < 0.001, and 514.5 ± 173.8 nmol/24 h vs. 249.4 ± 175.2 nmol/24 h, *p* < 0.001, respectively) and higher levels of PC after the DXM test (260.8 ± 158.8 nmol/L vs. 109.7 ± 82.7 nmol/L, *p* < 0.001). Regarding other biochemical parameters, in group B, we found significantly higher levels of total cholesterol (245.2 ± 42.5 mg/dL vs. 203.2 ± 39.9 mg/dL, *p* < 0.01), LDL cholesterol (150.5 ± 29.7 mg/dL vs. 121.5 ± 36.6 mg/dL, *p* < 0.01), and triglycerides (245.2 ± 42.5 mg/dL vs. 203.2 ± 39.9 mg/dL, *p* < 0.01) compared to group A.

None hormonal and biochemical differences were observed between two groups during follow-up. In both groups, compared to baseline evaluation, after hypercortisolism resolution we found a significant reduction of office systolic blood pressure (SBP) (group A 143 ± 20 mmHg vs. 137 ± 17 mmHg, *p* < 0.01; group B 146 ± 14 mmHg vs. 123 ± 19 mmHg, *p* < 0.01), office diastolic blood pressure (DBP) (group A 85 ± 12 mmHg vs. 82 ± 11 mmHg, *p* < 0.01; group B 93 ± 13 mmHg vs. 79 ± 13 mmHg, *p* < 0.02), and LDL cholesterol (group A 121.5 ± 36.6 mg/dL vs. 111.9 ± 33.9 mg/dL, *p* < 0.03; group B 150.5 ± 29.7 mg/dL vs. 116.0 ± 36.2 mg/dL, *p* < 0.02). Moreover, during follow-up, in group B, we have seen significant reduction of waist circumference compared to baseline (102.8 ± 8.7 cm vs. 90.8 ± 10.8 cm, *p* < 0.01) and total cholesterol (245.2 ± 42.5 mg/dL vs. 199.9 ± 30.0 mg/dL, *p* < 0.01).

## 4. Case Reports

### 4.1. Patient 1

In 2008, a 44-year-old woman was referred to our Unit for high blood pressure, moon face, excess hair growth, easy bruising, headache, and weight gain. She had a family history of diabetes type 2. The diagnosis of ACTH-dependent CS was confirmed by high serum cortisol with a lack of circadian rhythm and high serum ACTH. MRI showed a pituitary microadenoma. In June 2008, she underwent transsphenoidal surgery and the histological examination confirmed a basophil adenoma. Six months later, clinical and biochemical signs of cortisol excess significantly improved. Subsequently, in January 2009, patient reported fatigue, palpitations, and exophthalmos. A thyroid ultrasound showed whole increase of the gland. FT4 and FT3 levels and TSH receptor antibodies were high, whereas TSH was suppressed, suggesting diagnosis of Graves' disease. Patients underwent treatment with propranolol 40 mg/day and methimazole 15 mg/day, obtaining an acceptable control of the disease.

### 4.2. Patient 2

The second case was a 48-year-old woman referred for hypertension, moon face, buffalo hump, severe muscle wasting, and hair grown on the face. The diagnosis of ACTH-dependent CS was confirmed by hormonal analysis, and MRI detected a pituitary adenoma. She underwent transsphenoidal surgery in May 2007, and the histological examination confirmed microadenoma. Three months after surgery, her blood pressure and both PC and ACTH levels were normal.

However, after 8 months, she reported weakness and weight gain. The evaluation of thyroid function showed low FT4, TSH of 1.3 mIU/L, and positive TPO Ab (212 IU/mL, n.v. 1–50 IU/mL). Ultrasound imaging of the thyroid gland revealed a diffuse dyshomogeneous pattern, suggesting diagnosis of Hashimoto thyroiditis. She started therapy with levothyroxine 50 mcg once a day with clinical improvement.

### 4.3. Patient 3

In May 2013, a 52-year-old woman was referred with cortisol excess confirmed by high 24h-UFC values and an overnight 1 mg DXM test, bilateral adrenal gland lesions (right 26 mm; left 24 mm), and scintigraphic evidence of increased radionucleotide uptake by the left adrenal gland. The patient underwent left adrenalectomy, and histological evaluation confirmed an adrenal adenoma. During follow-up, both PC and 24h-UFC values were normal. In December 2013, she developed a skin disorder characterized by reddish or salmon-like, raised, scaly skin lesions in knees, buttocks crease, and ears. A dermatologist confirmed the psoriasis diagnosis and required a topical tacalcitol therapy.

### 4.4. Patient 4

A 38-year-old woman was diagnosed with CS, due to a 27 mm nodule of the right adrenal gland. She underwent right adrenalectomy, and histological examination confirmed an adrenal adenoma. Five months after biochemical and clinical resolution of hypercortisolism, she developed tremor of the hands, sweating, insomnia, weight loss, palpitations, anxiety, itch, and neck pain. Hyperthyroidism was suspected and confirmed by hormonal evaluation (TSH suppressed with elevated free fraction of T3 and T4 and the presence of anti-TSH receptor antibodies). Subsequently, methimazole 5 mg/day was administered with clinical improvement.

### 4.5. Patient 5

In January 2010, a 36-year-old woman with high blood pressure levels was evaluated in our specialized Unit. Plasma aldosterone, plasma renin activity, and 24h urinary metanephrines were normal. 24h-UFC excretion and PC at 8 am were higher with nonsuppression by an overnight 1 mg DXM test. MRI showed a left adrenal mass (40 mm). Three months later, left adrenalectomy was performed and histological examination confirmed an adrenal adenoma.

Two years after, the patient reported onset of oral aphthous ulceration in the hard palate, photosensitivity, symmetric arthralgias on wrists, fibromyalgia, and cutaneous vasculitis. Antinuclear antibodies (ANA) were positive, and p-Anti-Neutrophil Cytoplasmic Antibodies (ANCA) and c-ANCA were negative, confirming SLE. Hydroxychloroquine 200 mg twice per day was prescribed with subsequent clinical improvement.

### 4.6. Patient 6

A 45-year-old man was diagnosed with CS due to a right adrenal gland lesion (60 mm). Subsequently, the patient underwent right adrenalectomy and histological exam revealed multinodular hyperplasia of the adrenal gland. Three months after surgery, both PC and 24h-UFC values were normal and PC was suppressible by an overnight 1 mg DXM test. Nine months after surgery, he started to complain fatigue, sensitivity to cold, weight gain and reduced appetite, arthralgia, and myalgia. Thyroid function and antibodies were investigated, with a final diagnosis of hypothyroidism secondary to Hashimoto thyroiditis. Levothyroxine was administered with clinical improvement.

### 4.7. Patient 7

In 2011, a 56-year-old woman presented to our center reporting a high blood pressure level, fatigue, hirsutism, weight gain (body mass index 29 kg/cm^2^), and buffalo hump. Laboratory tests showed high PC, not suppressible after an overnight 1 mg DXM test, and high 24h-UFC and plasma ACTH levels. The diagnosis of ACTH-dependent CS was confirmed by MRI that showed a small left mass in the adenohypophysis. In January 2012, she underwent transsphenoidal surgery and the histological examination revealed a pituitary adenoma. Seven months later, she began to suffer from myalgia, arthralgia (shoulders, knees, wrists, and hands), headache, and long-lasting morning stiffness. Afterwards, she reported insidious swelling and pain of the first and second metacarpophalangeal (MCP) joints of the left hand. ANA were negative, whereas rheumatoid factor (RF) and C-reactive protein (CRP) were highly positive. Therefore, she was diagnosed a rheumatoid arthritis. She started therapy taking methotrexate once a week, folic acid 4 mg twice a week, and methylprednisolone 4 mg/day.

### 4.8. Patient 8

A hypertensive 62-year-old woman was admitted for the evaluation of an incidentally discovered right side adrenal mass. CT scan shown a 7 cm mass in the right adrenal gland, and after careful examination, the patient was diagnosed with subclinical hypercortisolism (SH). Adrenalectomy was performed in March 2013, and histopathological examination showed an adrenocortical adenoma. Significant symptoms and signs appeared 5 months later (bilateral ptosis, double vision, and proximal muscle weakness in the arms bilaterally); during specific evaluation, chest CT showed a solid tissue mass in the mediastinum. The diagnosis of myasthenia gravis was performed; thus, patient started on pyridostigmine 180 mg/day and prednisone 25 mg/day, with no evident benefit. Subsequently, she underwent a sternotomy with excision of the tumor, which histologically proved to be a type 2B thymoma. During the 1-year follow-up in our Unit, patient reported complete regression of myasthenic symptoms, with normal electrodiagnostic tests. She remained well on pyridostigmine 120 mg/day and antihypertensive therapy (amlodipine and bisoprolol) with good control of blood pressure [11].

### 4.9. Patient 9

A hypertensive 61-year-old woman with a right adrenal incidentaloma was studied in our specialized Unit. Both PC (at 8 a.m.) and 24h-UFC values were higher and not suppressible after an overnight 1 mg DXM test, suggesting CS diagnosis. In April 2013, she underwent right adrenalectomy and histological evaluation confirmed an adrenal adenoma. Four months later, she began to suffer from fatigue, mild headaches, bilateral aching, and stiffness of neck and shoulders. In September 2013, she went to emergency room, for right vision loss and severe headache. Ophthalmoscopy showed pale and swollen optic disc, dilated retinal veins, and several flame-shaped hemorrhages, such as an anterior ischemic optic neuropathy (AION). Intravenous methylprednisolone was administered. A cross-sectional biopsy showed transmural inflammation with mononuclear cells and giant cells infiltrating the media, compatible with temporal arteritis (Horton disease). After discharge, prednisolone p.o. 25 mg twice a day was administered with significant clinical improvement.

## 5. Discussion

CS is caused by chronic hypercortisolism resulting in a characteristic clinical phenotype and multisystem morbidity. The phenotypic characteristics of CS are well described, such as facial plethora, rounded face, decreased libido, thin skin, menstrual irregularity, hypertension, hirsutism, depression/emotional lability, glucose intolerance, weakness, and osteoporosis. Surgery is the treatment of choice for underlying causes of hypercortisolism, and pharmacological therapy has still a crucial role in inoperable patients [3–5].

Furthermore, it is known that in humans the inappropriate endogen secretion or exogen administration of cortisol may determine immunosuppression [12]. In fact, glucocorticoids (GCs) excess inhibits immune function by lymphoid tissue involution and lymphopenia as well as increased susceptibility to infections. On the other hand, GCs have a pivotal role in the anti-inflammatory functions [13]. Currently, few studies showed a link between surgical or medical resolution of endogenous hypercortisolism and the following exacerbation or development of an autoimmune diseases. We found in literature cases of exacerbating coeliac disease [14], rheumatoid arthritis [15–17], sarcoidosis [18–20], SLE [21], polymyalgia rheumatica [22], and thyroid diseases [9].

In a recent retrospective study, Tatsi et al. [23] studied a pediatric cohort of 127 children with CS; from these, 10 children were diagnosed newly onset autoimmune diseases after resolution of hypercortisolism (7.8% of prevalence). In particular, the types of autoimmune disorders were coeliac disease (no. 1), psoriasis (no. 1), Hashimoto thyroiditis (no. 1), Graves' disease (no. 1), optic neuritis (no. 2), skin vitiligo (no. 2), allergic rhinitis/asthma (no. 1), and neuropathy (no. 1).

Interestingly, it has been reported that patients with autoimmune diseases, compared to healthy subjects, have a reduced synthesis of GCs and altered CRP and cortisol/CRP ratio, in response to chronic inflammations such as polymyalgia rheumatica and giant cell arteritis [24, 25].

On the other hand, the cross-sectional study of Agha-Hosseini et al. [26] demonstrated that morning cortisol measurements were significantly higher in patients with Hashimoto thyroiditis than healthy patients, suggesting that in Hashimoto thyroiditis, cortisol excess (due to stress or specific disease) may downregulate hypothalamus and pituitary sensitivity to GCs, with the activation of the HPA axis and increased peripheral GC action. This pattern should lead to significant immune function changes, from a T helper (Th) 1 to a Th2 cytokine profile shift. In conclusion, in some autoimmune disease models, the Th2 immune response due to GCs action may have a protective role [27].

We have surgically treated overall patients with laparoscopic adrenalectomy (anterior or posterior approach), as suggested in literature [28, 29], with the absence of major postoperative complications.

In our study, patients with CS who developed autoimmune diseases after successful treatment show higher levels of PC (at rest and after the dexamethasone suppression test) and 24h-UFC values compared to the group of patients without autoimmune diseases, suggesting a possible trigger to higher endogen cortisol hyperproduction by underlying autoimmune disease, especially in patients affected by ACTH-dependent CS (present in 33% in group B).

Therefore, we believe that physiological levels of GCs are likely immunomodulatory rather than only immunosuppressive, resulting in the shift of cytokines from a prevalent proinflammatory to an anti-inflammatory pattern [30].

In our retrospective study, we evaluated 147 patients with CS; 109 patients underwent surgical treatment with definitive remission of excess of GCs and normalization of cortisol levels, during follow-up, and 9 (8.25%) patients showed an autoimmune disease. Most patients showed autoimmune thyroid diseases (Graves' disease and chronic Hashimoto thyroiditis). This result agrees with the previous study by Colao et al. that enrolled a series of patients with ACTH-dependent CS, specifically evaluating thyroid function in patients treated for CS, and showed increased risk of developing autoimmune thyroiditis after therapeutic resolution of hypercortisolism, with rising positivity for specific antibodies and occurrence of thyroiditis in 60% of treated patients [9]. The authors suggested an underlying abnormal immune response, in particular in those patients who were genetically disposed [10]. Therefore, the reduction of PC levels may developed by an immune response shift, characterized by increased levels of antithyroid antibodies [31]. Supporting this hypothesis we presented the case of our patient with myasthenia gravis (case No. 8) associated with positive acetylcholine receptors antibodies and a type 2B thymoma [11]. These clinical findings and our added reported cases tend to prove how the suppressive effect of exceeding GCs in CS modulates the immune system, with the possible autoimmune disease arising as consequence of a specific therapy [32].

In T cells, cortisol reduces the genetic expression of interleukin (IL)-2 and suppresses the function of T cell receptors and its downstream molecules (i.e., protein kinase Scr, lymphocyte-specific protein tyrosine kinase, inositol 1,4,5-triphosphate), through membrane-bound receptors [33]. GCs reduce the production of cytokines, chemokines, and arachidonic acid derivatives, the expression of Fc*ε* receptor I in mast cells, and the number of basophils and histamine production [34]. Low GCs concentrations activate macrophages (adhesion, chemotaxis, phagocytosis, and cytokine production), while high concentrations have an immunosuppressive effect [35].

Therefore, GCs excess certainly inhibits immune function. The improved GCs excess may, in several cases, restimulate individual immunity, triggering autoimmune diseases.

Endocrine and immune systems influence each other in a bidirectional fashion [36]. In fact, adrenal and sex hormones, as well as vitamin D, melatonin, and prolactin contribute importantly to the homeostasis of the immune system. Indeed, some of the steroidal hormone activities determine the inhibition or stimulation of immune system components, in both physiological (suppression of an unwanted response in pregnancy and stimulation of a protective response in infections) and pathological conditions.

Moreover, in the group of patients with the development of autoimmune diseases after CS treatment, other than higher levels of endogen overproduction of cortisol, we have found higher levels of total cholesterol, LDL cholesterol, and triglycerides, confirming the unfavorable metabolic effects of hypercortisolism, especially on the lipid pattern [37]. It is well described in literature that CS patients have higher cardiovascular mortality and morbidity due to several metabolic alterations, regarding glucose and lipid metabolism, improved after CS treatment [38]. As regards lipid metabolism, GCs can act through several mechanisms: increased expression of adipose triglyceride lipase and hormone-sensitive lipase (HSL) (involved in stored lipid breakdown in mature adipocytes), increased lipolysis by the cAMP-dependent activation of protein kinase A, impaired responsiveness to catecholamines and growth hormone (GH), increased expression/activity of lipoprotein lipase, higher neolipogenesis from substrates like glucose, and increased ectopic storage of lipids in the liver and skeletal muscle [37]. About it, in this study, the group of patients with higher levels of PC at diagnosis showed more significant reduction of waist circumference after resolution of hyperticolism.

Further evaluations are needed to understand complexity of the neuroendocrine network. We suggest to keep in mind, in the clinical management of CS, that an autoimmune disease which is silent during the acute phase of hypercortisolism may occur after the complete remission of the chronic exposure to high PC levels [15, 21].

In conclusion, we strongly believe that after treatment of CS, patients should be evaluated for the development of autoimmune disorders in order to obtain an early diagnosis and a proper treatment.

## Figures and Tables

**Table 1:** Etiology of Cushing's syndrome (CS), histology of endocrine disease, and immune disease in patients with treated CS and newly onset of autoimmune disease.

Case report	Sex	Etiology	Histology	Immune disease
1	F	AD	Pituitary adenoma	Graves' disease
2	F	AD	Pituitary adenoma	Hashimoto thyroiditis
3	F	AI	Adrenal adenoma	Psoriasis
4	F	AI	Adrenal adenoma	Graves' disease
5	F	AI	Adrenal hyperplasia	Systemic lupus erythematosus
6	M	AI	Adrenal hyperplasia	Hashimoto thyroiditis
7	F	AD	Pituitary *μ*-adenoma	Rheumatoid arthritis
8	F	AI	Adrenal adenoma	Myasthenia gravis
9	F	AI	Adrenal adenoma	Horton disease

M: male; F: female; AD: ACTH-dependent; AI: ACTH-independent.

**Table 2:** Demographic, anthropometric, hemodynamic, biochemical, and hormonal parameters at baseline and after a 24-month follow-up in patients with treated Cushing's syndrome (CS) compared with the group of Cushing's syndrome patients with posttreatment onset of autoimmune diseases (CS with AD).

	Baseline			24-month FU			*p*	
	CS (*n* = 100) A	CS with AD (*n* = 9) B	*p*	CS (*n* = 100) C	CS with AD (*n* = 9) D	*p*	A vs. C	B vs. D
Age (years)	61.0 ± 11.7	52.3 ± 12.7	0.03	63.1 ± 11.5	54.2 ± 12.8	0.03	ns	ns
Sex (M/F)	63/37	1/9		63/37	1/9			
BMI (kg/m^2^)	27.5 ± 4.6	29.2 ± 4.4	ns	27.5 ± 3.9	25.8 ± 5.6	ns	ns	ns
WC (cm)	98.4 ± 12.3	102.8 ± 8.7	ns	98.4 ± 14.5	90.8 ± 10.8	ns	ns	0.01
Office SBP (mmHg)	143 ± 20	146 ± 14	ns	137 ± 17	123 ± 19	ns	0.01	0.01
Office DBP (mmHg)	85 ± 12	93 ± 13	ns	82 ± 11	79 ± 13	ns	0.01	0.02
HR (bpm)	67 ± 12	75 ± 8	ns	66 ± 9	68 ± 14	ns	ns	ns
Creatinine (mg/dL)	0.84 ± 0.4	1.05 ± 1.06	ns	0.95 ± 0.35	1.19 ± 1.24	ns	ns	ns
Na^+^ (mmol/L)	142.9 ± 3.2	142.7 ± 2.4	ns	143.1 ± 2.6	141.4 ± 2.1	ns	ns	ns
K^+^ (mmol/L)	4.4 ± 0.5	3.9 ± 1.4	ns	4.4 ± 0.4	4.3 ± 0.2	ns	ns	ns
Calcium (mg/dL)	9.6 ± 0.5	9.0 ± 0.2	ns	9.6 ± 0.4	8.9 ± 0.4	ns	ns	ns
FG (mg/dL)	96 ± 24	92 ± 10	ns	96 ± 23	84 ± 16	ns	ns	ns
Uric acid (mg/dL)	5.2 ± 1.5	5.6 ± 1.1	ns	5.1 ± 1.3	5.5 ± 1.4	ns	ns	ns
TC (mg/dL)	203.2 ± 39.9	245.2 ± 42.5	0.01	196.7 ± 41.8	199.9 ± 30.0	ns	ns	0.01
HDL-C (mg/dL)	58.1 ± 17.4	51.8 ± 11.9	ns	61.2 ± 40.4	60.8 ± 15.9	ns	ns	ns
LDL-C (mg/dL)	121.5 ± 36.6	150.5 ± 29.7	0.01	111.9 ± 33.9	116.0 ± 36.2	ns	0.03	0.02
TGL (mg/dL)	113.4 ± 48.5	185.9 ± 112.8	0.01	124.9 ± 56.9	153.4 ± 112.3	ns	ns	ns
UFC (nmol/24 h)	249.4 ± 174.2	514.5 ± 173.8	0.001	155.2 ± 92.4	138.8 ± 83.5	ns	0.001	0.001
PC (nmol/L)	459.5 ± 160.3	818.1 ± 231.0	0.001	418.0 ± 118.0	373.5 ± 96.9	ns	0.05	0.001
PC DXM (nmol/L)	109.7 ± 82.7	260.8 ± 158.8	0.001	86.0 ± 25.1	40.0 ± 11.1	0.001	0.001	0.001

BMI: body mass index; WC: waist circumference; SBP: systolic blood pressure; DBP: diastolic blood pressure; HR: heart rate; Na^+^: serum sodium; K^+^: serum potassium; FG: fasting serum glucose; TC: total cholesterol; HDL-C: HDL cholesterol; LDL-C: LDL cholesterol; TGL: triglycerides; UFC: urinary free cortisol; PC: plasma cortisol; PC DXM: plasma cortisol postovernight 1 mg dexamethasone test.

## Data Availability

The data used to support the findings of this study are available from the corresponding author upon request.
